# Exploring Configuration Space and Path Space of Biomolecules Using Enhanced Sampling Techniques—Searching for Mechanism and Kinetics of Biomolecular Functions

**DOI:** 10.3390/ijms19103177

**Published:** 2018-10-15

**Authors:** Hiroshi Fujisaki, Kei Moritsugu, Yasuhiro Matsunaga

**Affiliations:** 1 Department of Physics, Nippon Medical School, 1-7-1 Kyonan-cho, Musashino, Tokyo 180-0023, Japan; 2 AMED-CREST, Japan Agency for Medical Research and Development, 1-1-5 Sendagi, Bunkyo-ku, Tokyo 113-8603, Japan; 3 Graduate School of Medical Life Science, Yokohama City University, 1-7-29 Suehiro-cho, Tsurumi-ku, Yokohama 230-0045, Japan; moritugu@yokohama-cu.ac.jp; 4 RIKEN Center for Computational Science, 7-1-26 Minatojima-minamimachi, Chuo-ku, Kobe, Hyogo 650-0047, Japan; ymatsunaga@riken.jp; 5 JST PRESTO, 4-1-8 Honcho, Kawaguchi, Saitama 332-0012, Japan

**Keywords:** molecular dynamics simulation, rare event, string method, multiscale enhanced sampling, weighted ensemble, multidrug transporter, Onsager–Machlup action

## Abstract

To understand functions of biomolecules such as proteins, not only structures but their conformational change and kinetics need to be characterized, but its atomistic details are hard to obtain both experimentally and computationally. Here, we review our recent computational studies using novel enhanced sampling techniques for conformational sampling of biomolecules and calculations of their kinetics. For efficiently characterizing the free energy landscape of a biomolecule, we introduce the multiscale enhanced sampling method, which uses a combined system of atomistic and coarse-grained models. Based on the idea of Hamiltonian replica exchange, we can recover the statistical properties of the atomistic model without any biases. We next introduce the string method as a path search method to calculate the minimum free energy pathways along a multidimensional curve in high dimensional space. Finally we introduce novel methods to calculate kinetics of biomolecules based on the ideas of path sampling: one is the Onsager–Machlup action method, and the other is the weighted ensemble method. Some applications of the above methods to biomolecular systems are also discussed and illustrated.

## 1. Introduction

A protein (except disordered proteins) usually has a definite three-dimensional structure (tertiary structure) formed by a sequence of amino-acids (primary structure), and, since there is a structure–function relationship, it is extremely important to clarify and characterize the 3D protein structure in atomistic detail [1]. Experimentally X-ray diffraction crystallography, nuclear magnetic resonance (NMR), and recently cryo-electron microscopy are usually employed to determine the protein structures, and (as of August 2018) 143840 structures have been resolved and stored in a protein data bank (PDB) [2,3]. Based on these structures, many chemical insights have been obtained. Such information continues to be utilized for the understanding of the biological function of a protein [1].

Even though the 3D structure is fully resolved with Ångström lengthscale, there is missing information on protein structural dynamics, which is a main focus of this review article. Of course, more and more advanced experimental techniques, including time-resolved X-ray diffraction crystallography [4], time-resolved IR [5], and UV-Raman [6] spectroscopy, have been developed, but their applications to protein systems are still limited. On the other hand, molecular dynamics (MD) simulation [7] among many computational methods has been also developed over the last few decades, and, with the advance of computer power and numerical algorithms, it has become an essential tool for computational chemists and experimentalists.

It is routine to simulate a protein in an explicit solvent or membrane with ∼100,000 atoms for ∼1 ms using MD simulations (if we can use the special-purpose computer Anton [8], it becomes ∼100 times faster), and the fluctuations of a protein around a naive structure might be fully characterized. However, difficulties arise due to *rare events* when we seek more biological consequences of protein dynamics. A rare event is a technical term [9,10], and researchers use it in two ways, as it has two different meanings: One is a less likely phenomenon, such as a huge earthquake, a terror attack, or a stock market crash, categorized as a very unlikely event. Corresponding to this definition in protein systems are metastable states that are less populated than a native state. The other is the rare transition between such unlikely events. This corresponds to the conformational change in protein systems because it crosses the (free) energy barrier *A* when conformational change occurs and is basically characterized by the Arrhenius law

(1) $$k∼\exp(-A/k_{B}T)$$

where *k* is the transition rate, and $k_{B}T$ is the temperature *T* multiplied by the Boltzmann constant $k_{B}$. Unfortunately, sampling these rare events in protein systems are basically not feasible using conventional MD simulations. This is because MD simulations tend to sample only a single basin around the initially starting structure and not the other basins. Of course, if we can wait a long time, other basins can be sampled but with a statistically insignificant amount. Metastable states and the conformational change of proteins are, however, the most essential elements in protein function, and we need to overcome this problem.

There are two strategies: One is to use coarse-grained (CG) models [11,12,13]. Because such CG models are simpler than the atomistic models with respect to computation of forces and energies, it is easier to calculate the dynamics and to sample the configuration space with a lower amount of computational resources. The drawbacks of using CG models are that constructing CG models can sometimes be cumbersome, and more importantly some detailed information is inevitably lost. In some cases, it is hard to extract time information such as kinetics from CG models.

The other is to use enhanced sampling techniques for atomistic models as described in this review. It is impossible to review all enhanced sampling methods (see [14] and the references therein), but there are basically two categories: one consists of models that modify the system parameters, the other of those that introduce artificial extended systems. The first category includes increasing the system temperature (e.g., temperature accelerated MD [15]) or boosting the potential bottoms (e.g., accelerated MD [16]). Since the protein dynamics is highly anisotropic [17], it is reasonable to utilize such “functional” directions [18] to modify the potential function (e.g., conformational flooding [19]). More general approaches are to modify the potential function so that the system feels (nearly) no force from such a modified potential, that is, the modified potential should be flat and guaranteed to easily sample the configuration space. The multicanonical method [20], the metadynamics method [21], and the adaptive biased force method [22] are such methods, which have been successfully used for many molecular systems. The second category includes extended ensemble methods such as replica exchange methods and their variations. In this type of method, we prepare many replicas with different parameters, such as temperatures or force constants, and exchange the parameters using Metropolis-type criterion. (In terms of using replicas with different initial conditions, the PaCS MD method recently devised by Harada and Kitao [23] is also considered as a variant of this category.) The temperature replica exchange method was first devised by Hukushima and Nemoto [24], introduced to the field of MD by Hansmann [25], Sugita, and Okamoto [26], and is now routinely used. Other important variants were introduced independently by Sugita, Kitao, and Okamoto [27] and Fukunishi, Watanabe, and Takada [28] and are called the multidimensional replica exchange method and the Hamiltonian replica exchange method, respectively. In these methods, we exchange parameters in the potential function such as force constants. These methods were further extended to multiscale systems using coupling between fine and CG degrees of freedom (DoF) by Moritsugu, Terada, and Kidera [29,30,31,32,33,34,35,36,37] as described below. (This method is also considered an extension of resolution replica exchange [38].)

Even if we could use the most sophisticated enhanced sampling techniques augmented by parallel computation, however, sampling the whole free energy landscape is impossible for large molecular systems [38]. The choice of collective variables (CVs) or order parameters can be another issue: if we cannot choose appropriate CVs, the convergence of the free energy calculation would be terribly slow [39,40]. This is a hard problem, but to remedy these difficulties, the string method, especially its extension to finite temperatures situations [41,42,43], is promising. Assuming that we know two metastable states and we are only concerned with the pathways connecting these metastable states, the string method is a powerful method to sample the pathways. We explain the basic principles of the method and several applications to biomolecular systems below.

Though many enhanced sampling techniques have been developed over the decades as mentioned above, and the mechanisms of reaction or conformational change for biomolecules can be clarified, there is still something missing: kinetics or dynamics of biomolecules. If the local equilibrium and several other assumptions hold, a reaction rate (or transition rate) can be estimated from the transition state theory (TST) or Kramers-type formulas [10,44,45,46]. The resulting rate formulas basically look like Equation (1), where *A* is the free energy barrier between a reactant and a transition state (more precisely we need to calculate a prefactor that depends on the shape of the potential energy surface and friction coefficient [10]). Hence, if we can accurately calculate *A*, then there seems to be no need to calculate the kinetics. Unfortunately this is wrong. Except for the practical difficulties of calculating *A*, the free energy landscape depends on the choice of CVs, and so does the reaction rate. As recently shown by Nakamura [47], if we do not carefully choose CVs, the free energy landscape as a function of CVs has a vague meaning.

As such, many researchers have been pursuing “direct” approaches to calculate the kinetics without the TST or Kramers-type formulas. One such example is the Markov State Model (MSM) [48,49,50,51], and, because of its simplicity for understanding and implementation, there have been many applications of this method. In the MSM, we prepare several initial states, which are assumed to be located between a reactant and a product state, run short-time MD simulations, and collect the resulting huge amount of trajectory data. Using some clustering algorithms or taking several dividing surfaces in some order parameter space for such data, we define so-called “micro” states in data space. We then count the numbers of transitions between “micro” states and construct a transition matrix. (In the conventional MSM, we need to introduce a lag time, whereas, in the milestoning [52], since the trajectory in order parameter space is assumed to be continuous, there is no need to introduce a lag time.) Manipulating the thus-obtained transition matrix, we can calculate the population in each state or, more importantly, the first passage time between such states. From the first passage time distribution, we can calculate the transition rates between microstates, which can be compared to experiments.

Though the MSM is a well-established and simple method, there are several assumptions which might hamper the justification for some applications. As such, some researchers have been developing different methods for kinetics, and path sampling methods are a general and sophisticated approach for this purpose [7,9,10]. In the path sampling approaches, a trajectory or “path” has a weight or probability as a whole, and based on such a weight we can devise a Monte Carlo move to sample huge path space. A good thing about this approach is that if we know a reactant and product, we can connect these states using the path sampling techniques without any biases, and in principle we can calculate any dynamical quantities (as well as equilibrium properties) with thus-obtained path ensembles. The Onsager–Machlup (OM) action method is such a path sampling method for overdamped Langevin dynamics, and we explain the basics of the OM method below [37,53]. On the other hand, the conventional path sampling methods use MD simulations, and, if the process is very slow, it is not efficient to sample path space with these methods. Hence, some modified types of path sampling techniques have been developed in the literature [10], and the weighted ensemble (WE) method is one such method [54]. We will introduce this method and discuss some applications to molecular systems below.

## 2. Multiscale Enhanced Sampling (MSES)

### 2.1. Overview of MSES

MSES is an enhanced sampling method for complex molecular systems, adopting an idea of multiscale simulations [29]. CG models are used for MSES because they have been successfully applied for extracting functionally relevant motions of biomolecules [11,12]. In MSES, the structural sampling of large proteins at atomic resolution is enhanced by coupling with accelerated dynamics of the associated CG model. Moritsugu and coworkers have developed various extensions of MSES [31,33] and have applied them to many protein systems of biological importance [30,32,34,35,36] as described below.

**Figure 1:**
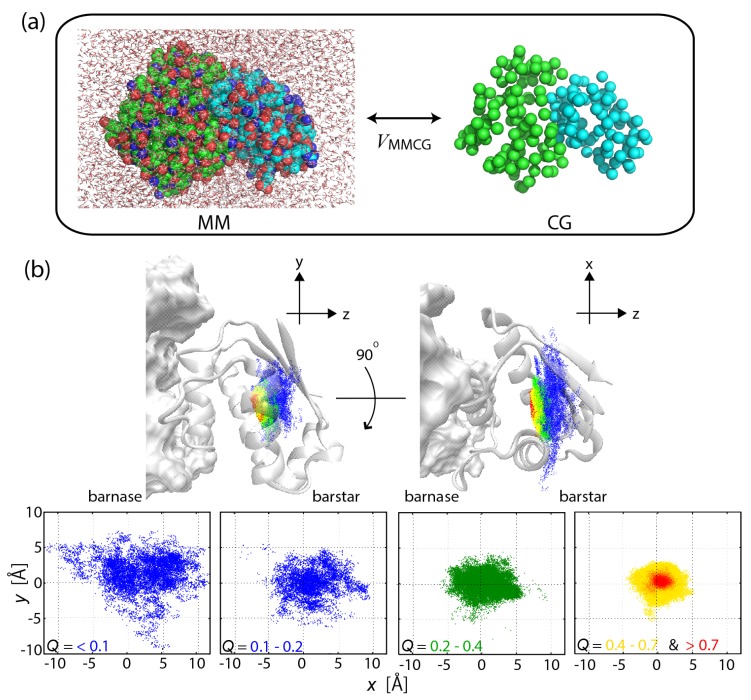
(**a**) Scheme of the multiscale enhanced sampling method. The structural sampling of MM is driven by CG model through the coupling $V_{\mathrm{MMCG}}$. Hamiltonian replica exchange [28] is adopted to eliminate the bias via $V_{\mathrm{MMCG}}$ and then to obtain the unbiased MM structural ensemble. (**b**) Funnel landscape of the protein–protein interaction for barnase–barstar complex, seen in a narrowing of the configurational space with increasing the fraction of native inter-molecular contacts formed (*Q*). For details, see [32].

In MSES, both a target physical system, e.g., an atomistic protein molecule in an explicit solvent (we call it MM), and the corresponding CG model are coupled. See Figure 1a. The potential energy of the multiscale system *V* is

(2) $$V\left(r_{\mathrm{MM}},r_{\mathrm{CG}},k_{\mathrm{MMCG}}\right)=V_{\mathrm{MM}}\left(r_{\mathrm{MM}}\right)+V_{\mathrm{CG}}\left(r_{\mathrm{CG}}\right)+k_{\mathrm{MMCG}}V_{\mathrm{MMCG}}\left(r_{\mathrm{MM}},r_{\mathrm{CG}}\right)$$

where $V_{\mathrm{MM}}$ and $V_{\mathrm{CG}}$ are the potential energies for MM and CG. The number of DoF in the CG systems is *M*, and it is therefore much smaller than that of the MM system *N*. The coupling term between the MM and CG systems $V_{\mathrm{MMCG}}$ is described by a harmonic constraint,

(3) $$\begin{array}{c} V_{\mathrm{MMCG}}={\left[\chi_{\mathrm{MM}}\left(r_{\mathrm{MM}}\right)-\chi_{\mathrm{CG}}\left(r_{\mathrm{CG}}\right)\right]}^{2} \end{array}$$

with the associated force constant $k_{\mathrm{MMCG}}$, where *K* component vector $\chi_{\mathrm{CG}}$ is arbitrarily defined by the use of the CG coordinates, and $\chi_{\mathrm{MM}}$ is the projection of $r_{\mathrm{MM}}$ onto the same *K*-dimensional space.

In order to obtain the structural ensemble of the intrinsic $V_{\mathrm{MM}}$, the bias through $V_{\mathrm{MMCG}}$ needs to be eliminated. For this purpose, the Hamiltonian replica exchange [28] is carried out, in which the replicated systems having various $k_{\mathrm{MMCG}}$ values from zero to a large value exchanges $k_{\mathrm{MMCG}}$ between the neighboring replicas. The exchange probability between replica *m* and replica *n* with different $k_{\mathrm{MMCG}}^{m}$ and $k_{\mathrm{MMCG}}^{n}$, derived so as to satisfy the detailed balance condition, is $p_{mn}=\min\left(1,\exp\left(\Delta_{mn}\right)\right)$ with

(4) $$\Delta_{mn}=\beta \left(k_{\mathrm{MMCG}}^{m}-k_{\mathrm{MMCG}}^{n}\right)\left[V_{\mathrm{MMCG}}\left(r_{\mathrm{MM}}^{m},r_{\mathrm{CG}}^{m}\right)-V_{\mathrm{MMCG}}\left(r_{\mathrm{MM}}^{n},r_{\mathrm{CG}}^{n}\right)\right]$$

where β = $1/k_{B}T$.

It is noted that the exchange probability is in proportion to the squared difference between $\chi_{\mathrm{MM}}$ and $\chi_{\mathrm{CG}}$, which are described by the *K*-dimensional space of the CG coordinates. Because of K∼M≪N, the smallness of $\Delta_{mn}$ or a high exchange probability $p_{mn}$ is assured irrespective of the number of the MM DoF *N*, leading to much higher scalability as compared with the conventional methods such as temperature replica exchange, where $p_{mn}$ is determined by the difference in the potential energy of MM (scaling up to $N^{2}$).

We can determine $V_{\mathrm{CG}}$ arbitrarily from prior knowledge or experimental data, depending on which subspace is targeted for enhanced sampling. More importantly, since K≫1, MSES allows a “predictive” structural sampling in that a distribution is roughly defined by CG and then refined through the MM force field. This kind of flexibility is advantageous over other methods using only a few predefined CVs.

### 2.2. MSES Extension Using Adiabatic Separation

In applying MSES to large protein systems including a number of explicit solvents, it is often the case that the force on the CG system from the MM system $-\partial V_{\mathrm{MMCG}}/\partial r_{\mathrm{CG}}$ overwhelms the CG intrinsic force $-\partial V_{\mathrm{CG}}/\partial r_{\mathrm{CG}}$, leading to the confinement in a stable basin where MM is strongly trapped. To overcome this problem, we have recently developed an extension by use of the approximation of adiabatic separation and the high CG temperature limit. Here we present a brief summary. See [33] for detail.

$r_{\mathrm{MM}}$, $r_{\mathrm{CG}}$ and $k_{\mathrm{MMCG}}$ are now considered as the independent variables of the joint distribution, $\rho \left(r_{MM},r_{CG},k_{\mathrm{MMCG}}\right)\propto \exp\left(-\beta V\right)$, with *V* defined in Equation (2): The unbiased MM ensemble is then obtained by extrapolating this ensemble to that with $k_{\mathrm{MMCG}}$ = 0. The joint distribution can be calculated by use of Gibbs sampling, i.e., by sampling $r_{\mathrm{MM}}$, $r_{\mathrm{CG}}$ and $k_{\mathrm{MMCG}}$ separately in an iterative manner with their conditional probabilities [55]. Suppose the CG mass being much larger than MM so that CG moves much slower than MM, while the CG temperature, 1/$\beta^{\prime }$, is much higher than that of MM; i.e., $\beta^{\prime }\ll \beta$. Under this approximation, named “adiabatic separation” [15,56,57], the conditional probabilities for $r_{\mathrm{MM}}$ and $r_{\mathrm{CG}}$ are written by

(5) $$\rho \left(r_{MM}\left|r_{CG},k_{\mathrm{MMCG}}\right.\right)\propto \exp\left(-\beta \left[V_{\mathrm{MM}}\left(r_{\mathrm{MM}}\right)+k_{\mathrm{MMCG}}V_{\mathrm{MMCG}}\left(r_{\mathrm{MM}},r_{\mathrm{CG}}\right)\right]\right)$$

(6) $$\rho \left(r_{CG}\left|k_{\mathrm{MMCG}}\right.\right)\propto \exp\left[-\beta^{\prime }V_{\mathrm{CG}}\left(r_{\mathrm{CG}}\right)\right]Z{\left(r_{\mathrm{CG}},k_{\mathrm{MMCG}}\right)}^{\beta^{\prime }/\beta }$$

where the partition function

(7) $$\begin{array}{c} Z\left(r_{\mathrm{CG}},k_{\mathrm{MMCG}}\right)\equiv \int dr_{MM}\exp\left(-\beta \left[V_{\mathrm{MM}}\left(r_{\mathrm{MM}}\right)+k_{\mathrm{MMCG}}V_{\mathrm{MMCG}}\left(r_{\mathrm{MM}},r_{\mathrm{CG}}\right)\right]\right) \end{array}$$

appears as the potential of mean force because of the slowness of CG relative to MM. It is straightforward that, by further taking the high CG temperature limit, or $\beta^{\prime }/\beta \to 0$, the CG conditional probability is simplified as

(8) $$\rho \left(r_{CG}\left|k_{\mathrm{MMCG}}\right.\right)\propto \exp\left[-\beta^{\prime }V_{\mathrm{CG}}\left(r_{\mathrm{CG}}\right)\right].$$

These derivations show that, while the sampling of $r_{\mathrm{MM}}$ is performed by the MD simulation under the $V_{\mathrm{MMCG}}$ constraint, the CG DoF $r_{\mathrm{CG}}$ is allowed to move freely, namely without the counterforce from MM, which results in the largest driving force for MM.

Finally, the sampling of $k_{\mathrm{MMCG}}$ is carried out by use of Markov chain Monte Carlo (MCMC) for discretized values of $k_{\mathrm{MMCG}}$. Here, we set the replicated systems of the MSES simulation to consist of many MMs, which are coupled with a single copy of CG, i.e., $r_{\mathrm{CG}}^{m}=r_{\mathrm{CG}}^{n}$. The exchange probability for the extended MSES then turns out to be the same as in the original MSES (see [33] for detail).

In summary, the simulation process consists of the following iteration: (1) the MD simulations of the replicated MM models and one CG model, and (2) the MCMC simulations in terms of $k_{\mathrm{MMCG}}$. The temperature and mass of the CG model must then be set to satisfy the conditions of adiabatic separation and $\beta^{\prime }/\beta \to 0$. To do this, e.g., the kinetic energies of the MM models need to be examined whether the energy flow from CG to MM is negligible [32,34].

### 2.3. MSES Applications to Biomolecular Systems

Applications have been performed for a broad range of complex protein systems of biological relevance, demonstrating the potential of MSES. Here, these were briefly reviewed by focusing on the CG model used, as the selection of $V_{\mathrm{CG}}$ might be critical for the success of the enhanced samplings, and on the number of replicas to show the scalability of MSES.

The order–disorder transition of an intrinsically disordered protein, sortase (a transpeptidase in Gram positive bacteria), was investigated by MSES [30]. The large-scale structural sampling of the disordered region was achieved by use of 20 replicas, indicating the usefulness of the flexibility for the recognition of the substrate peptide. Both the substrate-bound and -unbound structures were used to construct the CG model, which drives the order–disorder transition of the disordered region.

Protein–protein and protein–ligand interactions are the fundamental components in the interaction networks describing cellular processes such as signal transduction. The formation of the barnase–barstar complex was simulated on atomic detail by use of MSES, demonstrating a funnel-like energy landscape of the strong binder (with high affinity), which is formed through the specific side-chain interactions and the desolvation (Figure 1b) [32]. In contrast, the complex of di-ubiquitin (di-UB) and UB-binding domain (UBD) with relatively low affinity was found to have a subtle stability with an ensemble of highly dynamic structures, allowing dynamic recognition consisting of various binding modes [36]. In the applications, 12 and 16 replicas were used for barnase–barstar and di-UB/UBD complexes, respectively. To simulate the interaction process by the CG model, the Lennard–Jones potential was used for the protein–protein interactions, while the elastic network model [58] was applied for the rigidity of the intra-protein interactions.

The protein–ligand recognitions, especially in the case where the proteins undergo large structural changes between the open and closed forms, were also studied by use of MSES. In the CG model, the flexibility of the proteins was simulated by use of the double well model [59], which embeds the two open/closed structures, and the protein–ligand interactions, by the same Lennard–Jones potential as in the studies of protein–protein complexes. The full structural sampling of the glutamine binding to the glutamine binding protein, using 12 replicas for MSES, revealed the tight coupling between the protein structural change and the ligand interaction [34]. The derived energy landscape led to the determination of definite structural states, clarifying the dominant ligand interaction pathways in atomic detail. A pharmacological application to an important drug discovery target, glucokinase, was also carried out, demonstrating the drastic change of the energy landscapes depending on the glucose concentration [35]. This thermodynamics calculation, in combination with the weighted ensemble simulations (see Section 4.2), allowed the kinetics calculation of the millisecond-timescale structural transition that was found to be the same order as the experimental catalytic rate of glucokinase.

Recently, Hansmann and coworkers developed a new MSES method by adopting (1) a novel CG model using multiple Go-like potentials which can be switched like λ dynamics and (2) their replica-exchange-with-tunneling method for efficient MCMC [60,61]. This method was extensively applied to survey the structural dynamics of various amyloid fibrils using 16–32 replicas, such as the conversion of Aβ40 between parallel- and antiparallel-sheets [62], the formation and interconversion between fibril-like and barrel-like assemblies [63], and the transition of Aβ42 between the in-register and the out-of-register fibrils, and the barrel-shaped oligomers [64]. They also studied the fold-switch of the RfaH C-terminal domain between a double helix bundle and a β-barrel form [65]. Chen and coworkers also used the MSES method by adopting topology-based CG model, and simulated the folding/unfolding of kinase inducible domain of CREB using 16 replicas [66].

The general scheme of MSES will be applicable to broad classes of situations. For example, we adopted MSES to a path sampling method based on the Onsager–Machlup action, allowing a path ensemble to be efficiently sampled [37] (see also Section 4.1).

## 3. String Method

### 3.1. Overview of String Method

A number of different structures are frequently observed for a single protein in experiments, depending on their crystal or solution conditions (e.g., ligand-free, ligand-bound conditions). This kind of structural polymorphism generally implies an occurrence of structural changes in the cellular environment, which is often related to important biomolecular events (e.g., a transition from inactive to active state), so the mechanism of such a structural transition between observed structures attracts many researchers’ interests. The string method [41,42,43], described in this section, is a powerful approach to find a reasonable conformational pathway connecting two known structures. The method efficiently searches the most probable pathway and enables us to characterize the mechanism of the conformational change.

Suppose that we investigate conformational pathways of a transition from Reactant A to Product B. First, let us define a set of CVs $z\left(r\right)\in R^{N}$, where $r\in R^{3n}$ is the configuration of the system, and *n* is the number of atoms. These are projections of r to a low-dimensional, CG space, and *N* is usually much smaller than 3n. Typically, subsets of the Cartesian coordinates of protein atoms, the dihedral angles of backbone, or the distance between specific atoms are chosen as CVs. The free energy profile or effective potential energy that z(r) “feels” at $z^{*}$ in the CV space is given by

(9) $$F\left(z^{*}\right)=-k_{B}T\ln Z^{-1}\int \delta \left(z\left(r\right)-z^{*}\right)e^{-\beta U\left(r\right)}dr$$

where $Z=\int e^{-\beta U\left(r\right)}dr$ is a partition function, and U(r) is the potential energy of the system, and $\beta =1/k_{B}T$.

In the string method, we assume that most of the reactive trajectories that undergo conformational transitions from A to B, once projected in the CV space, go through a single thin tube (called the *reactive tube*). If the free energy barrier during the transition is much higher than $k_{B}T$, it was mathematically shown that the center of the reaction tube is well approximated by the minimum free energy pathway (MFEP) [41]. The geometry of the MFEP is represented by

(10) $${\left(M\left(z^{*}\right)\nabla F\left(z^{*}\right)\right)}^{⊥}=0$$

where the superscript ⊥ denotes the orthogonal component to the curve, $\nabla F(z^{*}\left(s\right))$ is a gradient of free energy, which is proportional to the mean force acting at $z^{*}$, and M(z) is a metric tensor which accounts for the curvilinear nature of the CVs, given by the following conditional expectation [41],

(11) $$M_{ij}\left(z^{*}\right)={\langle \sum_{k=1}^{3n}\frac{1}{m_{k}}\frac{\partial z_{i}\left(r\right)}{\partial r_{k}}\frac{\partial z_{j}\left(r\right)}{\partial r_{k}}\rangle }_{z\left(r\right)=z^{*}}.$$

The string method is an algorithm to efficiently search the MFEP using a set of replicated simulation systems instead of performing long brute-force simulations.

The MFEP, obtained by the string method, is able to capture the mechanism of the conformational transition because it allows us to determine the committor function [10,38,41]. The committor function, which is known to be *the best* reaction coordinate, is the probability that a trajectory initiated at an arbitrary point will reach first the Product B state without going back to the Reactant A state. This function allows us to derive various quantities of the transition, including the probability density of reactive trajectories, their probability current, and the rate of the reaction [67]. With properly chosen CVs, the MFEP is expected to be orthogonal to isocommittor surfaces [67]. Since the isocommittor surface of $\frac{1}{2}$ defines the transition state, the MFEP allows us to identify such a state. It is noted, however, that the accuracy of the MFEP in approximating the committor function crucially depends on the choice of CVs. This point will be discussed in the next subsection.

**Figure 2:**
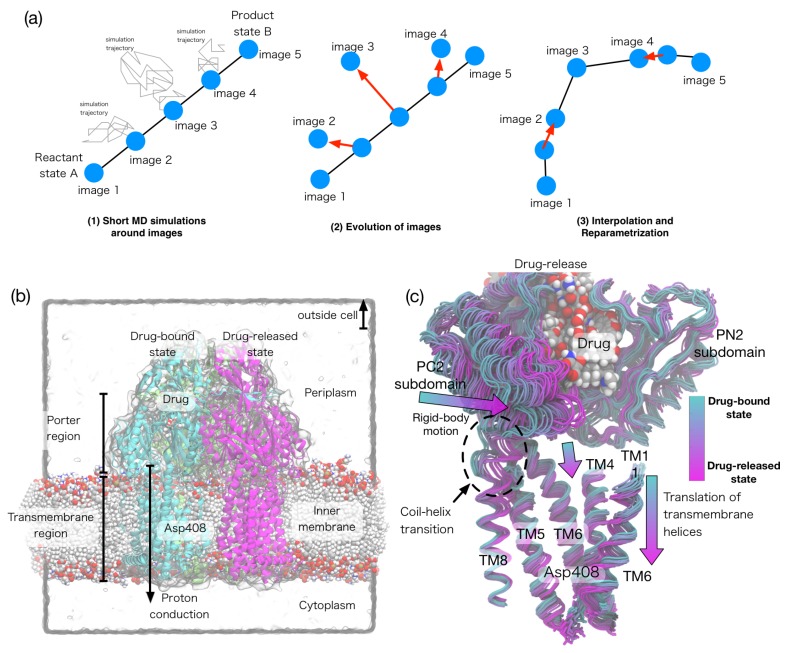
(**a**) Scheme of the string method algorithm; (**b**) Crystal structure of multidrug transporter AcrB embedded in the lipid bilayer; (**c**) Conformational transition (called the functional rotation) pathway, which links the proton binding/unbinding in the transmembrane region and the drug transportation in the periplasmic region. Modified and reprinted from [90].

Currently, there are three major algorithms in the string method: (i) the string method with mean forces [41], (ii) the on-the-fly string method [42], and (iii) the string method with swarms-of-trajectories [43]. In all of these methods, a pathway is represented by *m* discretized CV values (called *images*) connecting the A and B states (Figure 2a). In the mean force method, a short MD simulation samples conformations around each image with restraint potentials and computes a mean force and an average metric tensor. In the swarms-of-trajectories, a set of restraint-free simulations are initiated around each image and the average drift is computed. Then, each image is evolved using the calculated mean force and average metric tensor, (i.e., $M\left(r^{*}\right)\nabla F\left(r^{*}\right)$ in Equation (10)) or the mean drift. After the evolution of the images, a piecewise linear curve is interpolated through the images, and new images are distributed along this curve in an equidistant manner. This procedure is iterated until the pathway converges. The convergences of the pathway implies that the orthogonal component of $M\left(r^{*}\right)\nabla F\left(r^{*}\right)$ becomes zero for all images; thus, the converged pathway is shown to be the MFEP.

The string method is available in several MD software packages. The swarms-of-trajectories string method is available in NAMD [68] and the SANDER and PMEMD modules of AMBER. It is also available in GROMACS via Copernicus, a platform for parallel adaptive MD [69]. GENESIS supports the string method with mean-forces [70,71]. For a quantum string method [72] using the idea of centroid, PIMD code can be used [73].

### 3.2. Impact of the CV Choice on the Accuracy of Pathways

The choice of CVs is important because it determines not only the convergence rate of the string method calculation but also the accuracy of the MFEP in terms of the committor function. Are there then any principles for choosing better CVs? For large-scale conformational changes of proteins, such as open-to-close motions of multi-domain proteins, several choices of CVs have been proposed and demonstrated with a number of CV-based enhanced sampling methods. For example, Abrams and Vanden-Eijnden sampled conformational changes of the GroEL subunit and HIV-1 gp120 by using the temperature-accelerated molecular dynamics (TAMD) [74]. As CVs, they chose Cartesian coordinates of centers of contiguous subdomains, composed of 9 subdomains for GroEL and 14 subdomains for gp120. Vashisth and Brooks applied a potential energy bias in the direction of displacement calculated from the crystal structures and facilitated functional motions in their TAMD simulations [75]. In the context of the string method, Maragliano et al. showed, for accurate description of conformational transition of alanine-dipeptide, that four dihedral angles (rather than two) are required by evaluating committor functions [41]. Pan et al. showed that, through a comparison with brute-force simulations by Anton, root-mean-square deviations (RMSDs) of the flexible region are better CVs for capturing an accurate transition pathway of EGFR kinase rather than RMSDs of the entire molecule [76]. Recently, Matsunaga et al. examined how many CVs are required to capture the correct transition state during the open-to-close motion of Adenylate kinase’s CG model in the string method [77]. They tested various numbers of large amplitude principal components as CVs. Using the Bayesian statistics measure, they showed that the incorporation of local coordinates into CVs, which is possible in higher dimensional CV spaces, is important for capturing a reliable transition state. Taken altogether, in the string method, it is reasonable to choose flexible regions and multi-dimensional coordinates as putative CVs.

In order to obtain *a priori* putative CVs before starting a string method simulation, several systematic methods have been proposed. Ovchinnikov et al. proposed using targeted MD (TMD) simulation (where a harmonic restraint is used rather than the original holonomic constraint [78]) to identify a proper set of CVs to describe conformational changes [79]. Starting from the first trial set of CVs, they incrementally added candidate CVs until the heavy-atom RMSD between the final TMD simulation structures and the corresponding target structures was below a threshold value in both directions. Moradi et al. developed a set of computational protocols for efficient sampling of large-scale conformational changes of biomolecules [80,81]. Their protocols include a systematic CV assessment based on non-equilibrium work measurements with TMD or steered MD simulation [82].

### 3.3. String Method Applications to Biomolecular Systems

Thus far, the string method was successfully applied for finding the conformational transition pathways of various biomolecular systems, such as c-Src kinase [83], myosin VI [79], ion channels [84,85], 2-microglobulin [86], V1-ATPase [87], calcium pump [88], and membrane transporters [80,81]. Among them, purposes and results of the string method applications to Adenylate kinase [89] and the multidrug transporter AcrB [90] are described below, as illustrative examples.

Adenylate kinase is the best-studied biomolecule exhibiting a large conformational transition [91]. It is an enzyme which catalyzes the reversible phosphoryl transfer reaction: ATP+AMP↔2ADP. Its crystal structures suggests that, upon ligand binding, this enzyme undergoes a transition from the inactive open form to the catalytically competent closed structure. This transition is mediated by large-scale closure motions of three rigid-body domains (LID, AMPbd, and CORE domains). This functional open-to-close motion was investigated by the string method [89]. The MFEPs of the open-to-close transition were calculated by the string method using 20 largest-amplitude principal coordinates as CVs, under ligand-free and ligand-bound conditions. By comparing the two pathways, it was found that the LID domain was able to partially close without the ligand, while the closure of the AMPbd domain required the substrate binding. The transition state of the substrate bound form was identified as a highly specific binding state of the substrate to the AMPbd domain, and was validated by a committor test (see Section 3.1) with restraint-free MD simulations. These findings suggest that the interplay of the two different types of domain motion is an essential feature in the conformational transition of the enzyme.

The multidrug transporter AcrB transports a broad range of drugs out of the bacterial cell by means of the proton-motive force [92]. The drug transportation by transporters is one of the main causes of multidrug resistance in bacteria. Thus, understanding the mechanism of the drug transportation is important for the treatment of bacterial infections. The asymmetric crystal structure of trimeric AcrB suggests that a large conformational change in AcrB (called the functionally rotation) is coupled to the drug transport. Despite various supportive data from biochemical and simulation studies for this functional rotating mechanism, the link between the functional rotation and proton-motive force remained elusive. By calculating the MFEPs of the functional rotation for the complete AcrB trimer, the authors described the molecular basis behind the coupling between the functional rotation and the proton translocation [90]. Free energy calculations along the pathways showed that protonation of Asp408 in the transmembrane domain of the drug-bound protomer drives the functional rotation. The conformational pathway identifies vertical shear motions among several transmembrane helices, which regulate alternate access of water in the transmembrane as well as peristaltic motions that pump drugs in the periplasmic domain (Figure 2b,c).

## 4. Calculation of Kinetics for Biomolecules

### 4.1. Onsager–Machlup Action Method

The Onsager–Machlup (OM) action method is a genuine path sampling method based on the action principle. As is well known in classical mechanics, the Newton equation is derived from the action calculated by a Lagrangian from the least action principle. The same is true for the overdamped Langevin dynamics

(12) $$\frac{dx}{dt}=\frac{1}{\zeta }F\left(x\right)+\sqrt{2D}\eta \left(t\right)$$

where *x* is a system variable, F(x) is a force, ζ is a friction coefficient, $D=k_{B}T/\zeta$ is a diffusion coefficient, and η(t) is a Gaussian white noise, satisfying 〈η(t)η(0)〉=δ(t). In this case, we can define an action called the OM action as [38]

(13) $$S\left[x\left(t\right)\right]=\frac{1}{2}\int_{0}^{T}dt{\left(\frac{dx}{dt}-\frac{1}{\zeta }F\left(x\right)\right)}^{2}$$

where *T* is the total time for a numerical simulation. This is for the overdamped case, but for the underdamped case a similar action can be defined [93]. The important thing is that the path weight is determined by this action as

(14) $$P\left[x\left(t\right)\right]\propto e^{-S[x(t)]/2D}$$

and since this looks like a Boltzmann weight for a configuration, we can use all the gimmicks in equilibrium statistical mechanics such as replica exchange, reweighting, and so on here in path space. Of course, for numerical simulations, we use a discretized form of the action, and we can map the path space onto a connected beads system with some effective potential energy. The situation is very much similar to mapping a quantum system onto a connected classical beads system by the Chandler–Wolynes mapping [7].

The OM action and the other actions were combined with temperature replica exchange and used for sampling path space [53,94]. As mentioned above, MSES can be combined with the OM action to sample path space of a model polymer [37]. Some researchers used the OM action and the other actions for reweighting in path space [93,94,95,96,97]. By minimizing or optimizing the OM action and the other actions, we can obtain a “most probable” path, and such a strategy was used in [98,99].

### 4.2. Weighted Ensemble Method

**Figure 3:**
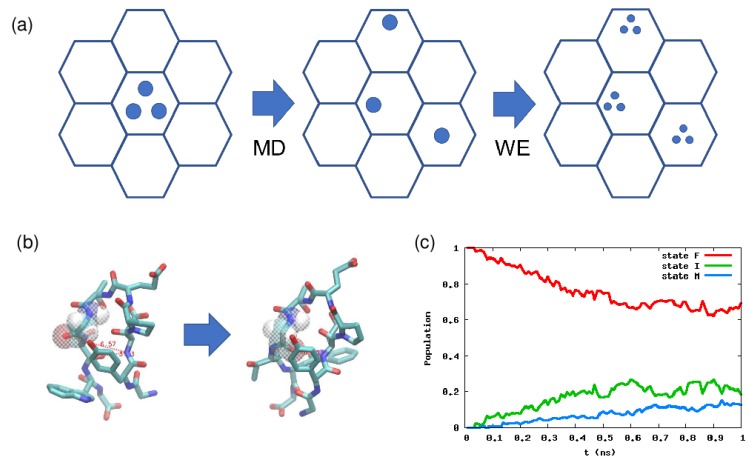
(**a**) Schematic picture of the weighted ensemble (WE) method. There are two phases: The MD phase and the WE phase. In the former, we run a normal MD simulation, and in the latter, we make multiple copies of a particle (trajectory), but we modify the particle weights accordingly. (**b**) Native and misfolded configurations of chignolin. (**c**) Population dynamics calculated by the WE method. Here the order parameters are the two hydrogen bond distances, which can discriminate the native and misfolded states. For details, see [101].

The weighted ensemble (WE) method, which was originally devised by Huber and Kim [100], and further elucidated by Zuckerman and coworkers [54], is a simple method for path sampling [7,10] in some CV space. The conventional path sampling methods (such as the OM action method mentioned above) utilize a weight for a path, whereas the WE method considers a weight for a configuration in CV space. The basic procedure of the WE method is as follows (see Figure 3a). For simplicity, we explain a nonequilibrium type simulation using the WE method. For the other types of the WE method, see [54] and the references therein.

We start from an initial state represented by several particles (trajectories). Let the number of the particles be *M*. Initially each particle has a weight 1/M, and summing them up leads to one. In a conventional setting, we divide the CV space into several cells (the hexagon in Figure 3a), and we check each cell every τ second in an MD simulation. We run a normal MD simulation using particles in an initial cell, and some particle might visit the other cells during τ. We then make multiple copies of such a particle in the other cells until the total number of particles becomes *M*. An important thing is that the total weight over all the cells should become 1 because of the conservation of probability. For example, if a particle with weight 1/M enters an empty cell, then M−1 particles should be generated and each particle should have a weight $1/M^{2}$.

If we further run the MD simulation, it is the case where particles enter a fully occupied cell. In such a case, we need to eliminate some of the particles such that the total number of that cell becomes *M*. We also need to modify the particle weights accordingly. Hence, this procedure can be regarded as a time evolution of a distribution function in CV space such as that calculated by the Fokker–Planck equation [38], and each CV variable is associated with a configuration (of biomolecules). Of course, if we prepare many particles in an initial state and run a normal MD simulation, and make a histogram in CV space, we basically get the same result. An important difference is that we divide the CV space into small cells and we basically monitor the transitions among such cells. If the distance between cells is “small,” then the transition is faster than the transition between a reactant and a product state that might be distant from each other. The acceleration of the transition is compensated by the smaller particle weights associated with the transition. This is the basic principle of the WE method and why this method works for the rare event problem.

The WE method has been applied to many systems, including the folding of a CG protein model, ligand binding, and chemical reaction networks [54]. Here, we show some examples we recently studied. One is the conformational change of a small peptide chignolin, which has a native state and a misfolded state (Figure 3b) [101]. In this case, two hydrogen bond distances are known to be good order parameters, so we divide this 2D space into several cells, and calculate the kinetics using the WE method starting from the native state. The result is shown in Figure 3c, and by linear fitting of the misfolded state population, we can estimate the transition time is ≃ 10 ns, which is similar to the mean first passage time calculated by non-Markov-type analysis and milestoning in [102]. For further details on how to calculate the rate constant or mean first passage time, see Chapter 10 in Zuckerman’s book [38].

The other example is the kinetics calculations of the structural change in glucokinase (GCK) [35]. The enhanced sampling using MSES (see Section 2) revealed that both the domain motion (between the open and closed forms) and the folding of an inter-domain helix (between the helical and coiled forms) are related to the regulation of the GCK function. Since the derived energy landscape also clarified the pathway of the structural change, as firstly the domain opening from the closed/helical (CH) to open/helical (OH) basins (path1) and secondly the helix collapse from the open/helical (OH) to open/coiled (OC) basins (path2), the WE calculations were carried out for the two paths, respectively, and the overall rate constant between the reactant and the product ($k_{\mathrm{CH}/\mathrm{OC}}$) was derived in combination with the three-basin kinetic model and the free energies of CH, OH, and OC. As described, the definition of the cells is essential for the success of the WE method: for path1, 22 cells were defined based on the first principal component representing the domain motion; for path2, the root mean square derivation from the helical form was used to define 52 equally divided cells. For the two paths, 50 ps MD simulations were carried out for *M* = 64 starting at each bin; 100 iterations were used (the total simulation time being 5 ns), and three WE runs were performed to make the error estimation for the derived rate constants. In the end, we obtained $k_{\mathrm{CH}/\mathrm{OC}}$ = 1.1 ms${}^{-1}$, indicating the GCK structural change in the timescale of milliseconds. This quantity results in a similar range to the experimental turnover rate of GCK phosphorylation, $k_{\mathrm{cat}}$ = 0.22 ms${}^{-1}$, suggesting the energy barrier between the closed/helical basin and the open/coiled basin as the origin of the GCK positive cooperativity.

## 5. Concluding Remarks

In this review article, after explaining the importance of enhanced sampling techniques for configuration and path space of biomolecules, we introduced and discussed the basic principles of the multiscale-enhanced sampling (MSES) method, the string method, the Onsager–Machlup (OM) action method, and the weighted ensemble method, all of which have been used by us for an enhanced sampling of biomolecules. We also illustrated several applications using the above-mentioned methods to some biomolecular systems. Our motivations for using these methods are their simplicity for understanding and implementation especially for large biomolecular systems. We hope that this review will facilitate further uses of these methods by the researchers in the field of computational protein dynamics.

Finally we mention our expectation of the future directions for computational protein dynamics.

(a) Larger molecular systems: Obviously, these enhanced sampling techniques will be used for much larger systems; recent foci in computational studies include on protein–protein, protein–DNA, and protein–RNA complexes, proteins in membranes, and proteins in crowded environments. The signaling pathways in a cell is a future target [103] and has already been studied in [32,36]. Though there have been several attempts to model atomistic details in a cell [104], multiscale-type methods such as MSES would be quite useful here. A signaling pathway represents sequential molecular processes, including proteins associations, dissociations, and associated chemical reactions, and sampling all molecular processes is not feasible, so the path sampling ideas such as the string method and the OM method should play a role. Multicellular dynamics could be another target [105]. It is quite unrealistic to model all atomistic details, so a CG model for multiple cells such as cellular Potts models might be combined with MD simulations to represent the associated molecular processes.
(b) More efficient methods: Recent advances in computational resources such as GPGPU and Anton [8] have been very promising, but it is still necessary to devise efficient numerical algorithms. In particular, for sampling dynamics or kinetics, we need to combine path sampling algorithms with conventional MD simulations, so the slowness of the latter would be a bottleneck. In materials science, hyperdynamics [106] is usually used to accelerate the barrier crossing processes, but it assumes the transition state theory (TST), which works best if the barrier height is much larger than $k_{B}T$. It is, however, not always the case for protein dynamics, and we need to use path sampling ideas to accelerate dynamics. It would also be promising to accelerate MD simulations using novel ideas of machine learning [107,108,109,110].
(c) Right CVs or reaction coordinates: Determining how to choose “right” CVs is always an issue, and the quality of the calculations heavily relies on this choice. We usually use intuitive and chemically “reasonable” variables, such as hydrogen bond distances, or geometrical measures, such as root mean square displacement (RMSD) from a reference structure and the radius of gyration ($R_{G}$). As mentioned above (Section 3.2), the committor function is *the best* reaction coordinate, and for its calculation we must know the transition states in advance, but it is not always the case. The string method can extract an importance multidimensional curve, connecting a reactant and product, but for diffusive pathways, the application of the string method encounters some difficulties. Hence, it has been a trend to borrow ideas from recently developed statistics methods to this field of protein dynamics. Principal component analysis (PCA) [17] has been used for extracting “large” functional motions of biomolecules as principal modes, but it is a linear and static analysis, and recently several other methods have been developed. Relaxation mode analysis (RMA) [111] or time-structure-based independent component analysis (tICA) [112] are such methods, and these methods can extract the slowest motions of biomolecules. Manifold learning techniques such as ISOMAP [113,114] and diffusion map [101,115,116] have been applied to a CG model or atomistic protein systems. Other machine learning techniques are also promising and await further application to biomolecular systems [107,108,109,110].

## Abbreviations

The following abbreviations are used in this manuscript:

MM	Molecular mechanics
CG	Coarse grained
CV	Collective variable
MSES	Multiscale enhanced sampling
MFEP	Minimum free energy path
OM	Onsager–Machlup
WE	Weighted ensemble
